# The comparison between short-term fertilization and conventional fertilization in *in vitro* fertilization treatment: a propensity score matching study

**DOI:** 10.3389/fendo.2026.1801741

**Published:** 2026-10-07

**Authors:** Dong Han, Xueying Kong, Jie Liu, Yan Zhou, Shiping Chen, Xiaomiao Zhao, Caixia Liu, Chaofan Zhang

**Affiliations:** 1 Department of Reproductive Medicine, Guangdong Provincial People’s Hospital, Guangdong Academy of Medical Sciences, Southern Medical University, Guangzhou, China; 2 Department of Clinical Laboratory, Dongguan Maternal and Child Health Hospital, Dongguan, China

**Keywords:** blastocyst, conventional fertilization, embryo, IVF, oocyte, short-term fertilization

## Abstract

Short-term IVF (S-IVF) is a fertilization approach to co-incubate the sperm and oocyte for a short duration and denude the cumulus cells afterwards. The S-IVF combined with early rescue intracytoplasmic sperm injection (ICSI) strategy can reduce the occurrence of both total fertilization failure (TFF) and the lower fertilization rate (<30%). However, there is still no consensus regarding the use of S-IVF. This study aimed to compare short-term *in vitro* fertilization (S-IVF) with conventional *in vitro* fertilization (C-IVF) in terms of fertilization, embryo developmental potential, and clinical outcomes. We used propensity score matching (PSM) to construct a 1:5 cohort of S-IVF and C-IVF cycles and compared fertilization, embryo developmental potential, and clinical outcomes between the two groups. We screened 68 S-IVF and 340 C-IVF patients (each in their first ART cycle—so cycle and patient numbers are identical) by baseline characteristics. The primary outcome was the fertilization rate, and the secondary outcomes were the pregnancy rate and abortion rate in cohort. The results showed that the C-IVF group had a higher fertilization rate [84% vs. 78%, *p* < 0.05], two-pronucleus (2PN) rate [67% vs. 60%, *p* < 0.05], and one-pronucleus (1PN) rate [5% vs. 3%, *p* < 0.05] compared with the S-IVF group. Furthermore, in the C-IVF group, the zero-pronucleus (0PN) rate was lower than in the S-IVF group (11% vs. 20%, mean difference -9 percentage points, 95% CI -12.5 to -5.5, *p* < 0.001). The good-quality blastocyst rate was numerically higher in the C-IVF group than in the S-IVF group, but the difference was not statistically significant. There was no significant difference in pregnancy rate or abortion rate in cohort between the two groups (early abortion rate among clinical pregnancies: 15.1% [14/93] in C-IVF versus 13.0% [3/23] in S-IVF, *p* = 1.000). In conclusion, C-IVF had a higher fertilization rate, 2PN rate, and 1PN rate but a lower 0PN rate compared with S-IVF, while there was no difference in embryo development and clinical outcomes.

## 1 Introduction

It is reported that 994,654 fresh ART cycles were performed in 76 countries in 2014, and conventional *in vitro* fertilization (C-IVF) was used for approximately 38% of patients (1). However, total fertilization failure occurred in approximately 5%–10% of C-IVF cycles, which may be caused by oocyte immaturity, failure of sperm–oocyte recognition, combination or penetration, and low sperm motility in asthenozoospermia patients.

Traditionally, fertilization failure in C-IVF was spotted in the fertilization check after 16–18 h of fertilization. Although intracytoplasmic sperm injection (ICSI) could rescue some of these oocytes, most had already missed their fertilization window, and the resulting embryos usually had poor fertilization rates (24%–48%) and low developmental potential (2, 3). In the case of total fertilization failure in C-IVF, short-term IVF (S-IVF) is applied in many IVF centers (4–7). This strategy is employed to co-incubate the sperm and oocyte for a short duration and denude the cumulus cells afterwards. The fertilization status can be observed by the presence of the second polar body (PB2). If the majority of oocytes did not show a PB2, embryologists could perform ICSI for early rescue (8).

Theoretically, S-IVF alone can reduce the occurrence of total fertilization failure (TFF) or a low fertilization rate (<30%) by allowing early detection of fertilization failure and timely intervention. Pongsuthirak (9) reported that this strategy enabled 79.2% of the oocytes from TFF cycles to fertilize. Besides that, some studies showed that S-IVF combined with early rescue ICSI can be conducted in cases of failed or lower fertilization with promising outcomes (10–12). Meanwhile, S-IVF has been claimed to increase the number of high-quality embryos as well as available embryos (13). However, other studies found that S-IVF would increase the polyspermic rate, leading to more multiple pronuclear embryos (14, 15). Moreover, most of these studies applied S-IVF in pre-selected patients with normal sperm characteristics but diagnosed with primary infertility for a long period. Their comparisons with normal C-IVF patients could lead to selection bias covering the potential effect of the S-IVF strategy.

Therefore, in this study, we used propensity score matching to construct a 1:5 cohort of S-IVF and C-IVF patients and compared their fertilization, embryo developmental potential, and clinical outcomes.

## 2 Methods and materials

### 2.1 Participants

In this study, we reviewed all patients under C-IVF or S-IVF treatment from January 2020 to August 2024. The inclusion criteria included the following: (1) the patient was under a gonadotropin-releasing hormone (GnRH) agonist or GnRH antagonist stimulation protocol, (2) all patients were in their first assisted reproductive technologies (ART) cycle with four or more retrieved oocytes, (3) the patient was planned for extended culture for blastocyst transfer, and (4) the method for sperm processing was gradient density. The choice of S-IVF was made by the treating clinician case by case, mainly based on the duration of infertility and the progressive rate (PR) in previous semen analysis. Cumulus cells were mechanically removed after 4 h of co-incubation of gametes (S-IVF group) from patients with unexplained infertility, primary infertility for more than 3 years, or where there was mild oligoasthenospermia (the total progressive motile sperm count in the post-preparation sample ≥2 million).

The exclusion criteria were as follows: for S-IVF cycles in which fewer than 50% of oocytes had extruded the second polar body at 6 h after insemination, rescue ICSI was performed, and these cycles were excluded from this study. No cycles between the two thresholds were managed differently, as a single unified criterion (PB2 <50% at 6 h) was applied consistently throughout the study period.

All of the patients had given informed consent for the study, and this study was approved by the Ethical Committee of Guangdong Provincial People’s Hospital for clinical research.

### 2.2 Stimulation protocol and oocyte retrieval

Patients in the cohort were under either GnRH agonist or GnRH antagonist protocol. The usage of the recombinant FSH was adjusted based on their age, ovarian reserve, E2 level, and follicles under beta-ultrasound. HCG trigger was performed when there were more than three follicles larger than 16 mm or two follicles larger than 18 mm. Oocyte retrieval was performed 36 h post-HCG trigger under beta-ultrasound guidance, and cumulus–oocyte complex (COC) was cultured with a culture medium covered with mineral oil at 37°C in a tri-gas (5% O_2_, 6%CO_2_, and 89% N_2_) incubator.

### 2.3 Semen collection

The male partner was prescribed sexual abstinence for 2–7 days. The semen was collected through masturbation and sent to the IVF lab within 1 h after ejaculation. The volume, viscosity, pH, concentration, and motility were recorded. Normal motility was defined as PR ≥32% or [PR + non-progressive rate (NR)] ≥40% based on the World Health Organization criteria (5th edition, 2010).

### 2.4 Fertilization and fertilization check

The semen sample was processed using density gradient centrifugation, and the sperm concentration was adjusted to 20,000–300,000 motile sperm per oocyte in a BD3037 dish. In S-IVF, the semen samples were added to the COC dish at 40 h post-HCG trigger, which was 2 h earlier than the C-IVF. Cumulus cell denudation was performed 4 h after fertilization in S-IVF. Then, the embryologist observed the presence of the second polar body. A single, prespecified criterion was used: if fewer than 50% of oocytes had extruded the second polar body at 6 h after insemination, the cycle was considered to have failed fertilization, and rescue ICSI was performed; these cycles were excluded from this study. For C-IVF, cumulus cell denudation was performed 16–18 h post-insemination. Fertilization checks in the C-IVF group were performed 16–18 h post-insemination, at which time the fertilization status of the S-IVF group was also checked. The presence of two pronuclei means that the oocyte was normally fertilized. The presence of one pronucleus was recorded as 1PN, while the presence of three or more nuclei was recorded as Multi-PN. If there was no pronucleus observed in the fertilization check, it was recorded as 0PN.

### 2.5 Embryo scoring

Embryos were scored on day 3 after fertilization. The grading system was based on the Istanbul consensus (16). In brief, a good-quality embryo (grade 1) was of <10% fragmentation, with all cell numbers and sizes of blastomeres coinciding with its development stage without multi-nucleation; a moderate-quality embryo (grade 2) was of 10%–25% fragmentation, with most cell numbers and sizes of blastomeres coinciding with its development stage without multi-nucleation; and a low-quality embryo (grade 3) was of >25% fragmentation, with cell numbers and sizes discrepant with its development stage and/or with multi-nucleation. The good-quality day 3 embryo originated from a 2PN zygote and scored in grade 1.

### 2.6 Pregnancy

Clinical pregnancy referred to one or more gestational sacs diagnosed by ultrasound examination, including normal intrauterine pregnancy, ectopic pregnancy, and intrauterine and ectopic concurrent pregnancy, as well as cases where only a gestational sac was visible without a fetal heart. Multiple gestational cysts were included in one case of clinical pregnancy. The clinical pregnancy rate was calculated by dividing the number of clinical pregnancy patients by the total number of patients in the matched cohort (per-cycle basis, including cycles with freeze-all, consistent with an intention-to-treat analysis). Outcomes of subsequent frozen-thawed embryo transfer cycles were outside the scope of this study. After confirming clinical pregnancy, spontaneous abortion within 12 weeks of gestation (excluding biochemical pregnancy) was defined as early abortion. The early abortion rate was calculated among clinical pregnancies (number of early abortions divided by the number of clinical pregnancies), which is the standard and preferred denominator. In addition, for transparency, we also report the abortion rate in cohort (number of early abortions divided by the total number of patients in the matched cohort).

### 2.7 Observation indicators

The observation indexes included fertilization rate, 2PN rate, 0PN rate, 0PN cleavage rate, 1PN rate, multi-pronuclei rate, D3 available embryo rate, D3 good-quality embryo rate, blastocyst formation rate, available blastocyst rate, good-quality blastocyst rate, pregnancy rate, and abortion rate in cohort.

### 2.8 Case matching

As the study focused on the laboratory outcomes after fertilization between the two groups, age, stimulation protocol, gonadotrophin (Gn) usage and duration, basal FSH, AMH, BMI, number of oocytes retrieved, infertility diagnosis, and male-factor variables (post preparation total progressive motile sperm count) were included in the propensity score model where available. A 1:5 nearest-neighbor propensity score matching (PSM) was then performed to eliminate the influence of baseline differences in this study. Covariate balance before and after matching was assessed using both *P*-values and standardized mean differences (SMD), with an absolute SMD <0.1 indicating adequate balance. Because the choice between S-IVF and C-IVF was made by the clinician, we acknowledge that residual confounding by indication cannot be fully excluded. The PSM was conducted by using R 4.2.1 software (The University of Auckland, Auckland City, New Zealand) with packages (17).

### 2.9 Statistics

Continuous variables were presented as mean ± standard deviation if they followed a normal distribution; otherwise, they were presented as median with interquartile range (IQR). The count data were represented as a percentage. Independent sample *t*-test or Mann–Whitney *U*-test (Wilcoxon rank-sum test) was used for group comparison in continuous variables, while chi-square test or Fisher’s exact test was used for group comparison in rates. To account for the clustering of multiple oocytes and embryos within the same patient, mixed-effects models or generalized estimating equations (GEE) were used where appropriate for oocyte- and embryo-level outcomes. For the primary fertility outcomes, the E-value was calculated using the R package “EValue” (18). Statistical analysis was conducted using R 4.2.1 software (The University of Auckland, Auckland City, New Zealand).

## 3 Results

### 3.1 Patients

As shown in **Figure 1**, a total of 605 cycles were initially screened. Of these, 21 S-IVF cycles were excluded because rescue ICSI was performed according to our prespecified criterion. Finally, a total of 584 cycles fulfilled our inclusion criteria from January 2020 to August 2024, comprising 68 S-IVF cycles and 516 C-IVF cycles. As all included patients were in their first ART cycle, the number of cycles equals the number of patients, and we therefore use “patients” and “cycles” interchangeably. We matched the S-IVF group with the C-IVF group in a 1:5 ratio under PSM and finally obtained 408 patients in our cohort (68 S-IVF patients and 340 C-IVF patients).

**Figure 1:**
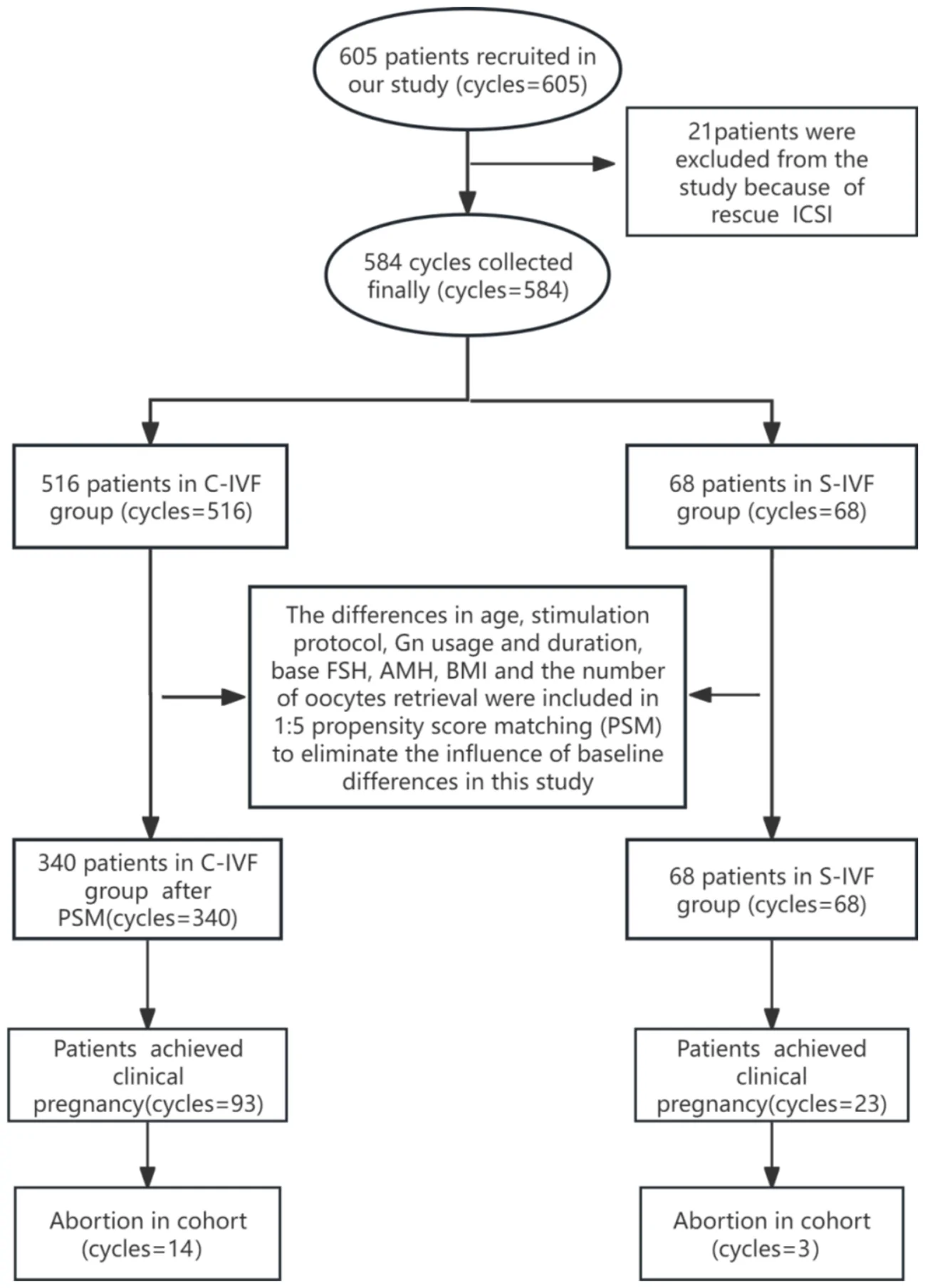
Consolidated standards of reporting trials (CONSORT) flowchart.

The baseline characteristics of our cohort are shown in **Table 1**. Before PSM, there were significant differences between the groups in female age, duration of infertility, number of oocytes retrieved, initial Gn usage, dosage of Gn, progressive sperm concentration, AMH, and E2 level. After PSM, the groups differed significantly only in the duration of infertility (*p* < 0.001) and post preparation total progressive motile sperm count (*p* < 0.05). That means female age, number of oocytes retrieved, initial Gn usage, dosage of Gn, AMH, and E2 level were similar between the groups after PSM. However, the duration of infertility and the progressive sperm concentration remained significantly different after matching. To address these residual imbalances, both variables were additionally adjusted for in the multivariable analysis, and balance was also assessed using standardized mean differences (SMD), with an absolute SMD <0.1 considered acceptable. In addition, a sensitivity analysis comparing the baseline characteristics of the 340 matched C-IVF patients with those of the 516 eligible C-IVF cycles confirmed that the matched sample was representative of the full eligible C-IVF population.

**Table 1:** Baseline characteristics of the short-term and conventional IVF groups.

		Before PSM			After PSM		
Parameter		C-IVF group	S-IVF group	*P*	C-IVF group	S-IVF group	*P*
Protocol (%)	GnRH-ant	172 (33.3%)	28 (41.2%)	0.310	123 (36.2%)	28 (41.2%)	0.353
	GnRH-a	344 (66.7%)	40 (58.8%)		217 (63.8%)	40 (58.8%)	
No. of patients		516	68		340	68	
Female age (years)		33.32 ± 4.80	31.28 ± 3.93	**0.001**	31.76 ± 4.45	31.28 ± 3.93	0.412
Duration of infertility (years)		3.58 ± 2.82	5.46 ± 2.69	**<0.001**	3.36 ± 2.59	5.46 ± 2.69	**<0.001**
No. of oocytes retrieved		13.40 ± 6.95	15.26 ± 7.63	**0.040**	14.64 ± 7.19	15.26 ± 7.63	0.518
Initial of Gn		183.62 ± 65.71	161.85 ± 53.88	**0.009**	163.66 ± 55.35	161.85 ± 53.88	0.805
Dosage of Gn		2,108.77 ± 788.25	1,845.76 ± 671.91	**0.009**	1,882.61 ± 668.63	1,845.76 ± 671.91	0.679
Duration of stimulation (day)		11.22 ± 2.66	11.26 ± 2.51	0.893	11.26 ± 2.78	11.26 ± 2.51	0.987
Post-preparation total progressive motile sperm count (×10^6^)		2.53 ± 0.93	2.15 ± 1.04	**0.002**	2.54 ± 0.93	2.15 ± 1.04	**0.002**
Basal FSH (mIU/mL)		7.39 ± 3.16	7.05 ± 2.62	0.389	7.13 ± 2.69	7.05 ± 2.62	0.815
AMH (ng/mL)		4.07 ± 3.03	5.45 ± 3.93	**0.001**	4.73 ± 3.28	5.45 ± 3.93	0.112
LH on HCG day (mIU/mL)		2.20 ± 2.63	2.24 ± 2.21	0.906	2.21 ± 2.67	2.24 ± 2.21	0.925
E2 on HCG day (pg/mL)		7.90 ± 0.67	8.12 ± 0.72	**0.014**	8.01 ± 0.56	8.12 ± 0.72	0.161
P on HCG day (ng/mL)		1.50 ± 2.79	1.51 ± 1.12	0.973	1.39 ± 1.12	1.51 ± 1.12	0.432
BMI of female (kg/m^2^)		21.85 ± 3.02	21.28 ± 3.54	0.156	21.40 ± 2.98	21.28 ± 3.54	0.778

Values are presented as mean ± SD unless otherwise noted. Bold emphasis indicates that the *P*-value is less than 0.05.

C-IVF, conventional *in vitro* fertilization; S-IVF, short-term *in vitro* fertilization; GnRH-ant, GnRH-antagonists; GnRH-a, GnRH agonist; Gn, gonadotrophin; FSH, follicle-stimulating hormone; AMH, anti-mullerian hormone; HCG, human chorionic gonadotropin; LH, luteinizing hormone; E2, estradiol; P, progesterone; BMI, body mass index.

### 3.2 Fertilization, embryo quality, and pregnancy outcomes

As shown in **Table 2**, there was no significant difference in the multi-pronuclei rate, day 3 good-quality embryo rate, blastocyst formation rate, available blastocyst rate, or pregnancy rate between the two groups. The C-IVF group had a higher fertilization rate [84% vs. 78%, mean difference of six percentage points, 95% CI 1.7 to 10.3, *p* < 0.05], two-pronucleus (2PN) rate [67% vs. 60%, mean difference of seven percentage points, 95% CI 2.1 to 11.9, *p* < 0.05], and one-pronucleus (1PN) rate [5% vs. 3%, mean difference of two percentage points, 95% CI 0.6 to 3.4, *p* < 0.05] compared with the S-IVF group. Furthermore, in the C-IVF group, the zero-pronucleus (0PN) rate was lower than in the S-IVF group (11% vs. 20%, *p* < 0.001). The good-quality blastocyst rate was numerically higher in the C-IVF group than in the S-IVF group, but the difference was not statistically significant. There was no significant difference in pregnancy rate or abortion rate in cohort between the two groups.

**Table 2:** Laboratory data and clinical outcomes of the short-term IVF group and the conventional IVF group after matching.

Parameter	C-IVF group	S-IVF group	*P*	E-values
No. of patients	340	68		
Fertilization rate (%)	84 ± 14	78 ± 17	**0.002**	2.22
Fertilization rate (2PN) (%)	67 ± 17	60 ± 19	**0.004**	2.15
Fertilization rate (1PN) (%)	5 ± 7	3 ± 5	**0.017**	2.09
Multi-PN fertilization rate (%)	6 ± 9	6 ± 8	0.857	1.18
Fertilization rate (0PN) (%)	11 ± 11	20 ± 14	**<0.001**	2.97
Cleavage rate of 0PN (%)	32 ± 41	37 ± 38	0.420	1.45
Good-quality embryo rate (%)	42 ± 24	38 ± 23	0.193	1.63
Blastocyst formation rate (%)	73 ± 22	73 ± 23	0.992	1.03
Available blastocyst rate (%)	75 ± 29	77 ± 27	0.564	1.36
Good-quality blastocyst rate (%)	42 ± 32	34 ± 31	0.058	1.84
Clinical pregnancy rate (%)	27.35% (93/340)	33.82% (23/68)	0.351	1.53
Abortion rate in cohort (%)	4.12% (14/340)	4.41% (3/68)	0.888	1.04
Early abortion rate (%)	15.1% (14/93)	13.0% (3/23)	1.000	–

Bold numbers indicate P values less than 0.05. Clinical pregnancy rate was calculated on a per-cycle basis (intention-to-treat), including cycles with freeze-all; outcomes of subsequent frozen-thawed transfers were not included.

C-IVF, conventional *in vitro* fertilization; S-IVF, short-term *in vitro* fertilization; 2PN, two pronuclei; 1PN, one pronucleus; 0PN, zero pronucleus; multi-PN, multi-pronuclei.

## 4 Discussion

Currently, there is no consensus on the use of S-IVF. In this study, we used propensity score matching (PSM) to compare S-IVF and C-IVF and provided a comprehensive view of their laboratory and clinical outcomes in a real-world setting.

The results of this study showed that the C-IVF group had a higher fertilization rate than the S-IVF group. This is consistent with the findings of Barraud-Lange (19) and Lundqvist (20) and may be explained by the higher progressive rate (PR) in the C-IVF group. The more progressive sperm in fertilization indicates a higher probability of penetration for the sperm into the cumulus–oocyte complex (COC) (21, 22), which would improve the fertilization rate. Moreover, the long co-incubation with cumulus cells in C-IVF could get more oocytes to develop into the MII stage, thus increasing the fertilization rate.

In terms of total fertilization, both of the 2PN and 1PN fertilization rates were significantly lower in S-IVF, but the 0PN rate was higher in this group. It may be because, as the S-IVF group was inseminated approximately 2 h earlier than the C-IVF group but both groups underwent pronuclear assessment at the same time the following morning, the pronuclear check in the S-IVF group occurred approximately 20–22 h after insemination, later than the recommended 16–18 h (16). As the optimal observation time for fertilization is 16–18 h after insemination (16), the relatively late pronuclear assessment in the S-IVF group might have missed the optimal pronuclear observation window, potentially accounting for the higher 0PN rate and the lower 2PN rate in this group. We therefore interpret the between-group differences in 0PN and 2PN rates with caution. In this study, there was no difference in the multi-pronuclei rate between the two groups, which was in agreement with the results of Wei (23) and Zhang (24).

However, Dai (10) and Xue (25) found that the removal of the cumulus cells 4 h post-insemination would increase the multi-pronuclei rate. The function of the cumulus cell was to aid oocyte maturation by facilitating fertilization (26–29). Xiong (30) suggested that the early removal of cumulus cells could compromise the oocyte’s defense against polyspermy. In our study, the 68 S-IVF patients and their matched cohort received either the GnRH agonist or GnRH antagonist protocol, with no statistically significant difference in protocol distribution between the groups, implying that the differences in 0PN, 1PN, and 2PN rates were not associated with the stimulation protocols. Furthermore, the combined 2PN and 0PN rate was high in each group, which indirectly indicates that oocyte maturation was satisfactory.

For the embryo development outcomes and the clinical outcome, there was no difference between the two groups. Although the S-IVF group had a lower overall fertilization rate, this did not translate into a lower clinical pregnancy rate. This is plausible because a single good-quality blastocyst is sufficient to achieve pregnancy, and the similar blastocyst formation and good-quality blastocyst rates between the two groups suggest that, once an embryo was obtained, its developmental competence and implantation potential were comparable. As we matched the S-IVF with C-IVF in basic characteristics (age and BMI), baseline hormone (FSH and AMH), and controlled ovarian hyperstimulation (stimulation protocol, Gn usage, and duration), the intervention differences were mainly in the fertilization method. Oocyte synchronization and maturation, as well as patient demographics, were expected to be balanced between the groups. Therefore, embryo development and clinical outcomes were similar in both groups, which is consistent with the results reported by Kong (31) and He (8).

Although we matched the S-IVF with C-IVF in a 1:5 ratio, the sample size of the S-IVF group was small and, as with any retrospective cohort study, residual and unmeasured confounding could not be fully excluded. We, therefore, performed an E-value analysis (32) to assess the robustness of our findings to unmeasured confounding. The E-values for the fertilization rate, 1PN, 2PN, and 0PN differences were all greater than 2, indicating that an unmeasured confounder would need to be associated with both the treatment allocation and the outcome by a risk ratio of more than 2 to fully explain away the observed associations. Our results, therefore, appear robust to unmeasured confounding. Nevertheless, given that the observed differences in 0PN and 2PN rates may partly reflect differences in the timing of pronuclear assessment and that rescue–ICSI cycles were excluded, the between-group differences in fertilization outcomes should be interpreted with appropriate caution. Several further limitations should be acknowledged. First, patients with conditions that can affect fertility and pregnancy outcomes, such as polycystic ovary syndrome (PCOS), endometriosis, and adenomyosis, were not excluded or adjusted for, although their distribution was balanced by matching on the available baseline characteristics. Second, the 1:5 matching ratio resulted in an uneven group size (68 versus 340), which may reduce statistical efficiency; nevertheless, all group comparisons used unpaired tests appropriate for independent samples. Third, because only the 340 matched C-IVF patients were analyzed, we performed a sensitivity analysis comparing the baseline characteristics of the matched 340 C-IVF patients with those of the 516 eligible C-IVF cycles, which confirmed that the matched sample was representative of the full eligible C-IVF population. Fourth, the choice of S-IVF was based on clinician judgment, which may introduce confounding by indication; although PSM reduced this risk, residual confounding cannot be excluded. Fifth, mechanistic explanations in this retrospective study remain speculative, and experimental studies would be required to confirm the underlying biological mechanisms.

In conclusion, by using PSM to match the S-IVF and C-IVF groups, we obtained balanced groups that allowed a fair comparison of the two treatments. The C-IVF had a higher fertilization rate, 2PN rate, and 1PN rate, but a lower 0PN rate, compared with S-IVF, while there was no difference in embryo development and clinical outcomes. However, the sample size was small and a larger prospective matched trial is needed for further confirmation.

## Data Availability

The original contributions presented in the study are included in the article/supplementary material. Further inquiries can be directed to the corresponding author.
